# Automated Sensor Node Malicious Activity Detection with Explainability Analysis

**DOI:** 10.3390/s24123712

**Published:** 2024-06-07

**Authors:** Md Zubair, Helge Janicke, Ahmad Mohsin, Leandros Maglaras, Iqbal H. Sarker

**Affiliations:** 1 Department of Computer Science and Engineering, Chittagong University of Engineering and Technology, Chittagong 4349, Bangladesh; zubairhossain773@gmail.com; 2 Centre for Securing Digital Futures, Edith Cowan University, Perth, WA 6027, Australia; h.janicke@ecu.edu.au (H.J.); a.mohsin@ecu.edu.au (A.M.); 3 School of Computing, Edinburgh Napier University, Edinburgh EH14 1DJ, UK; l.maglaras@napier.ac.uk

**Keywords:** cybersecurity, malicious node detection, wireless sensor node, data balancing, ensemble learning, explainability analysis

## Abstract

Cybersecurity has become a major concern in the modern world due to our heavy reliance on cyber systems. Advanced automated systems utilize many sensors for intelligent decision-making, and any malicious activity of these sensors could potentially lead to a system-wide collapse. To ensure safety and security, it is essential to have a reliable system that can automatically detect and prevent any malicious activity, and modern detection systems are created based on machine learning (ML) models. Most often, the dataset generated from the sensor node for detecting malicious activity is highly imbalanced because the Malicious class is significantly fewer than the Non-Malicious class. To address these issues, we proposed a hybrid data balancing technique in combination with a Cluster-based Under Sampling and Synthetic Minority Oversampling Technique (SMOTE). We have also proposed an ensemble machine learning model that outperforms other standard ML models, achieving 99.7% accuracy. Additionally, we have identified the critical features that pose security risks to the sensor nodes with extensive explainability analysis of our proposed machine learning model. In brief, we have explored a hybrid data balancing method, developed a robust ensemble machine learning model for detecting malicious sensor nodes, and conducted a thorough analysis of the model’s explainability.

## 1. Introduction

**Figure 1:**
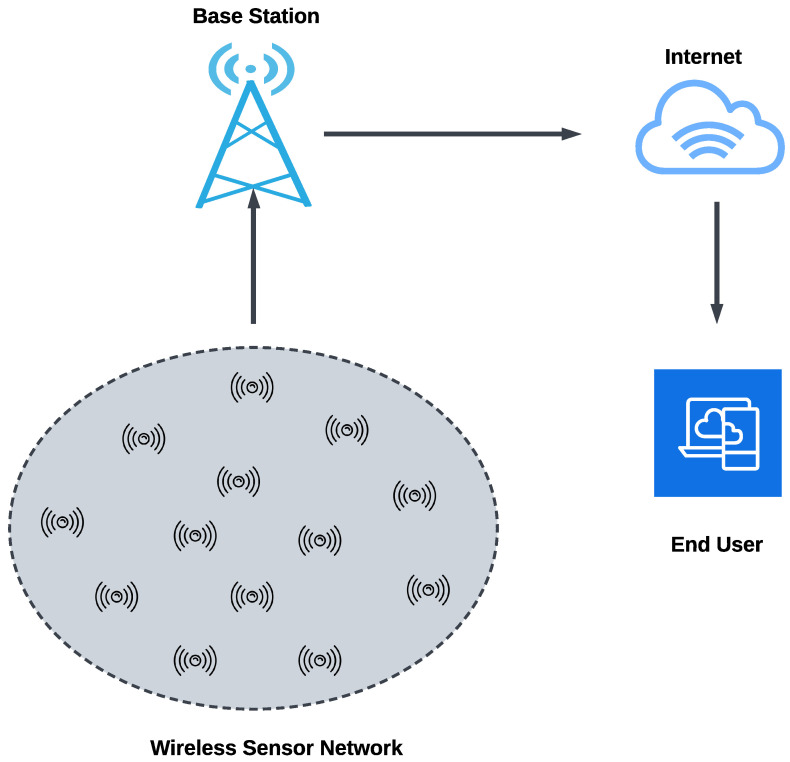
Basic wireless sensor network. Wireless sensors are connected to a base station and end-users communicate with the sensors via the internet through the base station [5]. Malicious activity may happen at any stage of the wireless communication network.

We live in a digital world where Cyber-Physical Systems (CPS) are an integral part of our lives. The recent Industrial Revolution was fueled by the need for advancement in these systems [1]. In CPS, physical devices are connected to the cyber world through wireless communication, mainly via the internet, and controlled remotely [2]. Most of these systems are autonomous and use sensors to perceive environmental conditions. Sensor technologies are being used in various sectors, including healthcare, smart agriculture, and transportation systems [3]. Sensors transmit data through wireless communication channels. Figure 1 demonstrates the basic working principle of wireless sensor communication. However, this characteristic poses security issues, making it easy for an adversary to attack the system. Attackers often try to intrude into the sensor network to pose a security threat and cause significant damage to the CPS. In dealing with cyber attackers attempting to breach the security of the network, human intervention is usually limited [4]. Therefore, automated security protection is highly required to safeguard the sensor network.

As the internet becomes more widespread, attackers have easier access to information and modern technology. This enables them to constantly adopt new technologies and change their attack methods, rendering rule-based cybersecurity protection systems ineffective. This is especially true for systems driven by CPS and consisting of various sensor networks. Artificial intelligence, especially machine learning (ML), is the most effective method to tackle the issue, enabling autonomous and dynamic protection systems [6]. Machine learning models are data-driven and most of their performance is dependent on the training dataset. Even a robust ML model may underperform when exposed to low-quality data. Quality and balanced data are crucial for training ML models. Typically, cybersecurity datasets are highly imbalanced, with rare positive classes (e.g., malicious, attacked).

In this research, we have worked to detect malicious sensor nodes in order to safeguard CPS-driven systems. We have carefully addressed all the issues explained earlier and used the dataset of malicious sensor nodes for our entire experiment [7]. The dataset was highly imbalanced, so we proposed a hybrid data balancing technique that combines undersampling and oversampling methods. The processed dataset fits ML models better than other state-of-the-art data balancing techniques. We have trained multiple classification ML models such as Logistic Regression (LR), Gaussian Naive Bayes (GNB), Support Vector Machine (SVM), Decision Tree (DT), Random Forest (RF), XGBoost (XGB), Artificial Neural Network (ANN), 1D Convolutional Neural Network (1D CNN), Recurrent Neural Network (RNN) and Long Short-Term Memory network (LSTM) [8]. Finally, we have proposed an ensemble method that outperforms other related models. In addition, we have showcased the explainability analysis of our suggested model. This analysis is essential as it helps us to comprehend the ways in which security breaches occur, spot weaknesses, and develop better response strategies. Our work includes the following significant contributions:

– We have proposed a systematic hybrid data balancing technique using cluster undersampling and the SMOTE oversampling method.
– We have proposed an ensemble learning method that outperforms other state-of-the-art ML models in detecting malicious nodes.
– We have also conducted a detailed explainability analysis of our model to determine which features are contributing to specific decisions and why.

In our research article, we outline various research outcomes and procedures in Section 2. We discuss the proposed data balancing method and data preprocessing strategy in Section 3. In Section 4, we present the overall methodology. Experimental results are discussed in Section 5 and Section 6 shows the detailed explainability of our proposed model, while the discussion, future work, and limitations are described in Section 7. Finally, in Section 8, we conclude our work.

## 2. Related Work

Cybersecurity has become a major concern for researchers around the world as it poses a significant threat to modern society [9]. There are various reasons why cybersecurity can be breached, such as malicious activity, phishing, intrusion, spam, ransomware, and spyware, among others, all perpetrated by cyber attackers. Artificial intelligence is currently the most promising field to safeguard the cyber world against these threats [10]. Industries and critical infrastructure are undergoing automation and digitization. Most often these modern technologies are controlled by sensors. It is important to note that any malicious activity in the sensor network can result in significant damage. Generally, two types of cyber threat detection systems are used to protect the system: signature-based and automated ML-based systems. However, ML-based systems are considered more robust than signature-based techniques, as per a comparison study [11]. Some of the recent works have been included in this section to offer insights into the previous research endeavors in the domain.

Researchers [12] proposed a random forest-based machine learning model to identify military and environmental wireless communication networks. They used the KDD Cup 99 dataset and balanced the training dataset with the oversampling technique, which slightly improved the accuracy level. An imbalanced data handling technique named ’Difficult Set Sampling Technique (DSSTE)’ [13] was established. In this model, the dataset was divided into two difficult and two easy sets, and at the last step, the difficult minority data was augmented to balance the minority class. This proposed architecture was evaluated on NSL-KDD and CSE-CIC-IDS2018 datasets for wireless sensor intrusion detection. Moreover, Unnamed Aerial Vehicles (UAVs) are not free from intruders as these use a large number of wireless sensors. Whelan et al. used a one-class classifier to detect intrusions in the UAVs, which required only non-anomalous sample data [14]. They considered GPS Spoofing attacks throughout their work.

An imbalanced dataset might affect the classification problem in cybersecurity. Generally, the quantity of normal data is larger than attack data, which leads to a high classification error. The authors of the paper [15] used a class-balancing technique for intrusion detection datasets. They used the K-Nearest Neighbour (KNN) algorithm for undersampling the nonintrusive data and the tabular auxiliary classifier generative adversarial networks model (TACGAN) for oversampling intrusive data. The final dataset was created with a combination of undersampling and oversampling data. Y. Fu et al. [16] proposed a deep learning model for network intrusion detection (DLNID), combining attention mechanism and Bi-LSTM network. Adaptive synthetic sampling (ADASYN) was created for sample expansion of minority class samples.

Deep learning models are not always suitable for dealing with the malicious activity of wireless networks. The work [17] shows the Hidden Markov Model (HMM) and Gaussian Mixture Model (GMM) stochastic assumptions outperform other machine learning models. Additionally, they also work on their own dataset. Another group of researchers worked on intrusion detection of IoT sensors. They mainly focused on the Gaussian Nave Bayes (GNB) and Stochastic Gradient Descent (SGD) algorithms to tackle the intrusions [18]. S. Salmi et al. [19] explained the reason for the usage of a wide range of sensors in different fields and why these are vulnerable to security issues. They analyzed the efficiency of the existing techniques to detect DoS attacks in wireless sensor networks and found that the Convolutional Neural Network (CNN) was the best model for detection. To perform the experiment, they used the publicly available WSN-DS dataset [20]. Microgrids of renewable energy also use a large number of sensors to operate the distributed system. Any attack on the sensor could potentially damage the energy sector. So, a group of researchers used a Recurrent Neural Network (RNN) model to detect the attack. Finally, they validate the model with MATLAB and OPAL-RT [21].

Blockchain is another ground-breaking technique to safeguard the cyber world, especially, for transactions or other data register cases. Machine Learning (ML) classifiers are integrated into the system to detect malicious activity. When the ML model detects a node as malicious, the blockchain declines its entity, while for legitimate nodes, the blockchain accepts the data. In a blockchain-based security research project for distributed data storage, security measures were implemented using Base Stations (BSs) and Cluster Heads (CHs) for node registration. Additionally, a machine learning classifier called Histogram Gradient Boost (HGB) on BSs was used to distinguish between malicious and legitimate nodes, rescinding registration for the former and storing data of the latter in an Interplanetary File System (IPFS) [22]. H. Hasan et al. [23] proposed an algorithm named XGBCLUS for detecting anomalies in the blockchain payment transaction, which is an ensemble method of learning. They also explained which features contribute to capacity and analyzed the model’s explainability.

## 3. Dataset Overview and Data Balancing

The reliability of data-centric models depends on the quality of the dataset. Low-quality data produces inaccurate results [24]. It is of utmost importance to carefully craft and preprocess datasets. Throughout the paper, we utilized the SensorNetGuard Dataset (Malicious Sensor Node Detection) [7]. In this section, we will demonstrate the overview of the dataset and explain our proposed data balancing technique in detail.

### 3.1. Dataset Overview

In cyberattack datasets, it is common that the positive class (attacked data) is rare compared to the negative class. We also observed similar characteristics in the SensorNetGuard dataset. Table 1 represents the number of Malicious (487) and Non-Malicious (9513) nodes.

**Figure 2:**
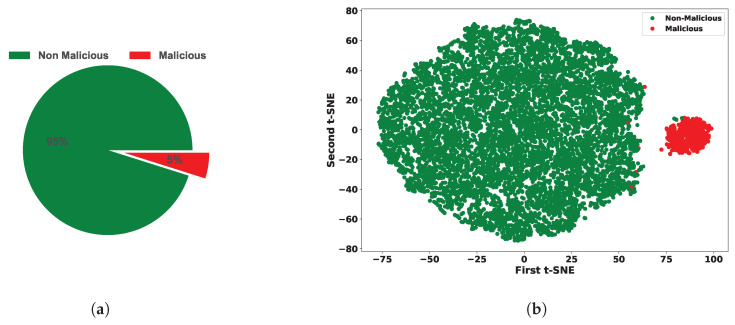
(**a**) Ratio of malicious and non-malicious instances in the dataset and (**b**) distribution of Malicious and Non-Malicious sensor nodes data with a 2D t-SNE plot.

So, we have found the dataset is highly imbalanced where the malicious class is only 5% and the non-malicious class is 95%. Figure 2a depicts the class ratio with a pie chart.

**Features and Example of the Dataset** 

The dataset contains twenty-one features that are divided into eight categories. These features represent various metrics of sensors and are listed in a Table 2.

The dataset is complete and does not contain any missing values. A few data instances are shown in Table 3. The dataset contains numerical data, except for the *IP Address* column, which contains object-type data.

### 3.2. Data Visualization

Data visualization enables a better understanding and provides insights into datasets. Understanding how data are distributed and identifying areas for improvement is crucial for optimizing the dataset.

#### 3.2.1. Data Distribution

We cannot visualize objects with more than three dimensions. However, our data has 21 features, which equates to 21 dimensions. To visualize the data, we need to reduce the dimensionality to 2 or 3. For better representation, we have used the best dimensionality reduction algorithm t-SNE, and visualize the data in two dimensions (2D) [25]. Figure 2b displays a 2D plot of our dataset, depicting malicious and non-malicious data points. Although there is a loss of information due to dimensionality reduction, the plot provides an intuitive understanding of the dataset.

#### 3.2.2. Data Distribution of the Features

**Figure 3:**
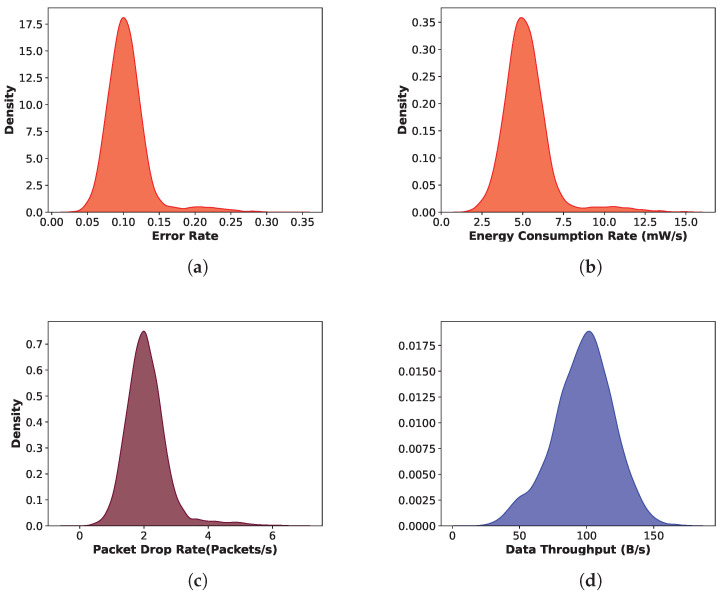
Distribution of the most important features (**a**) Error Rate, (**b**) Energy Consumption Rate, (**c**) Packet Drop Rate, and (**d**) Data Throughput for sensor node.

We have plotted the density distribution of the four important features according to the description of the feature selection in Section 3.3. Figure 3 depicts the density distribution of the features *Error Rate, Energy Consumption Rate, Packet Drop Rate, and Data Throughput.*

Figure 3a,b represent the data density distribution of Error Rate and Energy Consumption Rate respectively and both of them are right-skewed. Slight skewness of the Packet Drop Rate is visible in Figure 3c and the Data Throughput feature is slightly left-skewed, which is represented in Figure 3d. In case of feature selection, skewed data hold more information than the normally distributed data.

### 3.3. Feature Selection

Feature selection is a critical stage in machine learning models. We have dropped the General Metrics *(Node ID, Timestamp, and IP Address)*, as these values are unique and do not have any effect on the target variable. In the selection process of other features, we have determined the dependency of the dependent variables on the independent (target) variable. All of our dependent features are numerical, but our output feature is categorical. So, we have selected the ANOVA (Analysis of Variance) F-value for feature selection [26]. We calculate the ANOVA F-value for each feature and the target variable. In feature selection, higher F-values indicate that the features are more informative for predicting the target variable.

**Figure 4:**
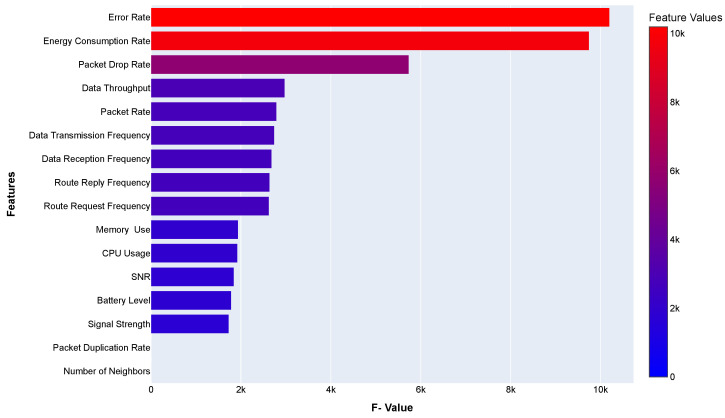
ANOVA F-values for each feature of the sensor node dataset.

The horizontal bar plot in Figure 4 represents the F-value for each feature and is sorted in descending order from top to bottom. The first two features’ F-values are the highest, and Packet Duplication Rate and Number of Neighbours features are the lowest. So, we have eliminated the lowest F-value features.

### 3.4. Data Balancing

Class imbalance is a common issue in cyberattack datasets, as there are usually only a few instances from the attack class. On the other hand, machine learning models fail to train their parameters properly if the training dataset is highly imbalanced. So, we have proposed a data balancing technique to address the issue. In this subsection, we will discuss the proposed method of data balancing. The entire precise procedure is given in Algorithm 1.

In the proposed balancing technique, we under-sample the majority class and over-sample the minority class. Therefore, we split the majority and minority classes first. For the majority and minority classes, we have proposed cluster undersampling and SMOTE-based oversampling techniques, respectively. Finally, we merge the undersampled and oversampled data to create a new balanced training dataset. Algorithm 1 is designed to represent the step-by-step procedure of the proposed balancing technique.
**Algorithm 1** Data Balancing Algorithm1:**Input**: An imbalanced dataset D.2:**Output**: Balanced Dataset.3:**procedure** Proposed Hybrid Data Balancing Model(Input)4:    Identify the Majority and Minority classes.5:    **if** class = Majority **then**6:        Drop the class level of the majority class.7:        Use the Elbow method to find the optimum cluster number (K).8:        Apply K-means or other clustering algorithms to create K number of clusters.9:        Follow stratified undersampling technique to extract data from each cluster.10:       Keep the sample size (50–60)% of the entire dataset.11:    **else if** class = Minority **then**12:       Apply Synthetic Minority Over-sampling Technique (SMOTE) for oversampling.13:       Keep the sample size (40–50)% of the entire dataset.14:    **end if**15:    Merge the cluster under-sampled and SMOTE over-sampled data.16:**end procedure**

#### 3.4.1. Cluster Undersampling

According to Algorithm 1, if the class is the majority, we systematically undersample it. We follow the steps below for undersampling.

**Figure 5:**
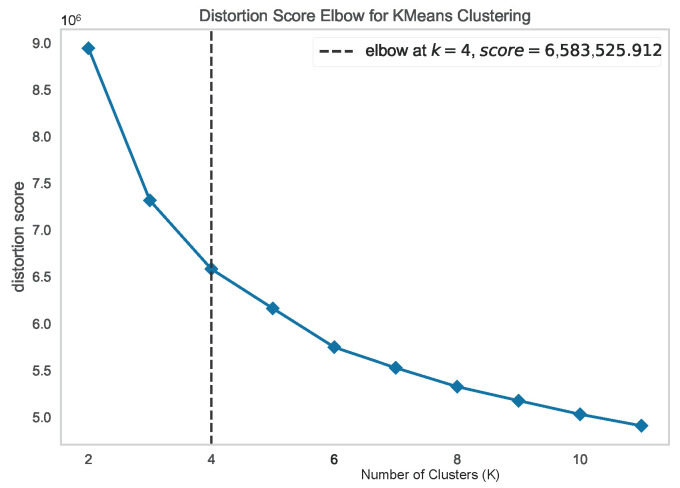
Optimum number of clusters of the Majority (Non-Malicious) class using the elbow method, where the value of K in the *X*-axis represents the number of clusters.

**Figure 6:**
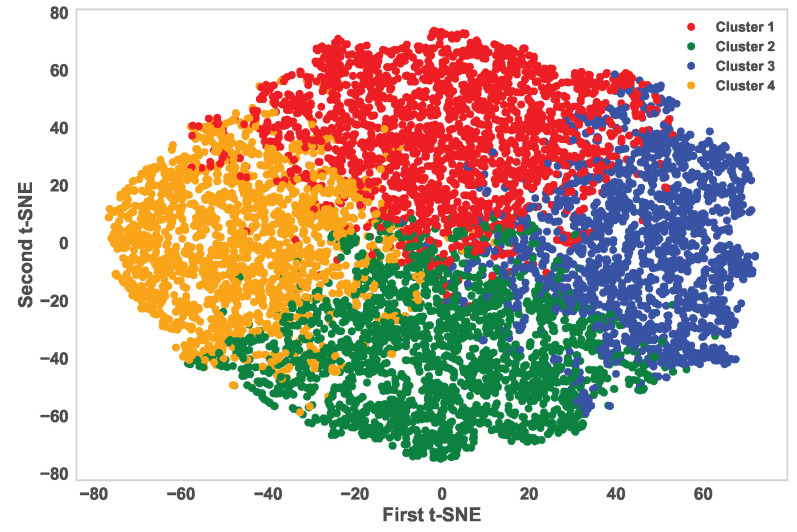
Four clusters with two dimensions t-SNE scatter plot. Each color (red, green, blue, and orange) represents an individual cluster.

- **Number of Optimum Clusters:** We need to find the optimum number of clusters for the majority class. The elbow method is used to identify the optimum cluster numbers [27]. For the malicious sensor node dataset, the majority class is the non-malicious class. So, we have dropped the class label and applied the elbow method for the identification of the optimum cluster number. Figure 5 shows the implementation of the elbow method for finding the optimum cluster number. The *X*-axis of the figure represents the number of clusters denoted with K and the *Y*-axis represents the Distortion score (average squared distance from the clusters centroids) for the respective number of clusters. According to the elbow method, the optimum cluster number for the non-malicious class is 4 (K = 4). We create four clusters using the K-means clustering algorithm in the next step.
- **Apply K-means for Cluster Creation:** K-means is a simple and one of the most widely used state-of-the-art unsupervised machine learning algorithms [28]. We have utilized the algorithm for cluster creation and created clusters with the majority (non-malicious) class data, which has helped us to unleash the underlying categories inside the class. Figure 6 demonstrates the distribution of the four clusters of the majority class with a 2D t-SNE scatter plot.
- **Systematic Data Extraction:** We extracted data from each cluster in a stratified manner to reduce information loss. This approach ensures that we have data from each category.
- **Size of Undersampled Data:** Our dataset is a binary classification dataset. A binary dataset is considered balanced when the proportion of datapoints in each class (positive and negative) is approximately equal, usually around 50%. As most of the data in our dataset are non-malicious, we have kept the sample size (50–60)%.

#### 3.4.2. SOMOTE Oversampling

Our dataset contains only 5% malicious data, shown in Figure 2a. So, we have observed a huge imbalance in the dataset and need to oversample the data to eliminate the ML model’s overfitting problem. Compared to other oversampling techniques, SMOTE is the best model to handle the issue [29].

- **Apply SMOTE Oversampling:** In our dataset, the malicious class is the minority class consisting of only 5% of the entire dataset. To resolve the overfitting and underfitting problems, we have applied SMOTE oversampling to generate synthetic data of the minority class.
- **Size of Oversampling Data:** When creating synthetic data, there are always possibilities of information loss and noise. So, we have kept the sample size of the oversampled minority class to (40–50)%.

#### 3.4.3. Merging the Oversampled and Undersampled Data

**Figure 7:**
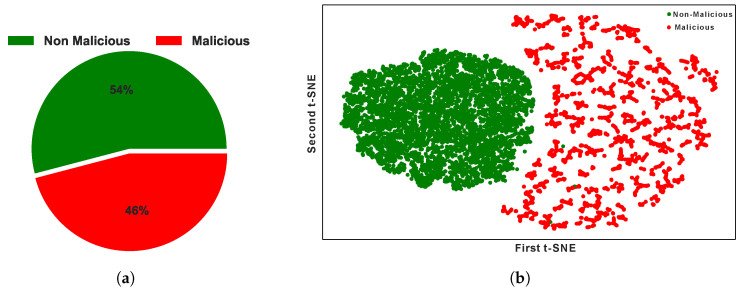
(**a**) Ratio of malicious and non-malicious instances in the balanced dataset and (**b**) Distribution of Malicious and Non-Malicious sensor nodes in the balanced dataset with a 2D t-SNE plot.

In the last two subsections, we have created two sample datasets (with Majority class undersampling and Minority class oversampling). Now, merging the datasets is the final step of our data balancing technique. Therefore, we have merged the undersampled and oversampled data to create the final balanced dataset. After balancing the class imbalance problem, we have found the ratio of the classes, as shown in Figure 7a. The data distribution of our new balanced dataset is represented in Figure 7b.

All the steps are compiled in the proposed Algorithm 1. This balanced dataset improves the quality of the training data significantly, which helps to create efficient ML models. Though we have applied our proposed method to the SensorNetGuard dataset [7], it is applicable to solve the imbalance problem of any dataset.

## 4. Methodology

**Figure 8:**
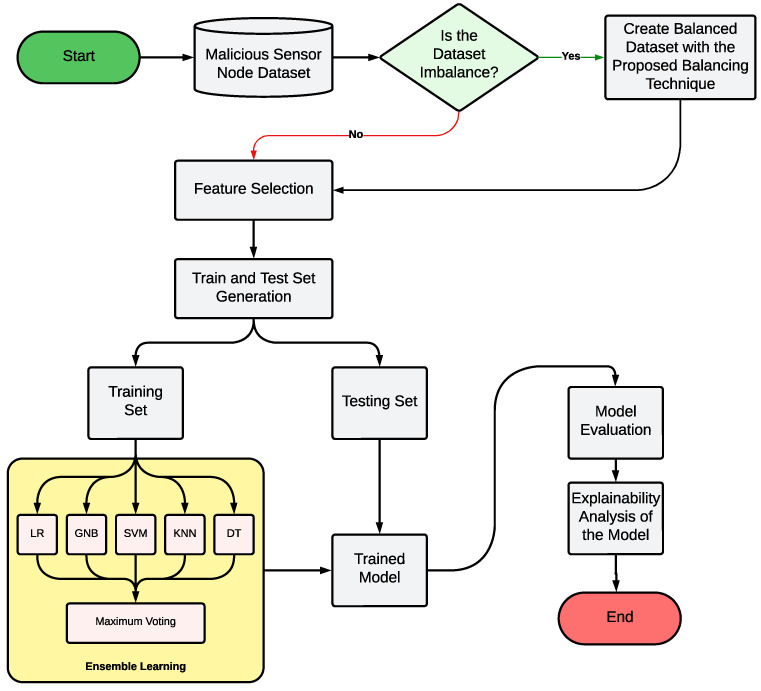
Detailed workflow of the overall procedure of our proposed malicious sensor node detection technique.

In this section, we will provide a detailed explanation of the overall procedure involved in our proposed technique. *The procedure can be broken down into three stages: (1) dataset collection and preprocessing, which includes feature selection and data balancing, (2) ML model creation, and (3) explainability analysis technique.* This section includes all the core concepts for our proposed model. Figure 8 represents the precise overview of our proposed model.

### 4.1. Dataset Collection and Preprocessing

Data represent the first step for any ML model. We have used the SensorNetGuard dataset [7], which is a recent dataset consisting of malicious and non-malicious sensor nodes. According to the workflow shown in Figure 8, we have checked whether our dataset is balanced or imbalanced. Our dataset is imbalanced, as represented in the Figure 2. So, we have applied the proposed data balancing technique, mentioned in Section 3.4. (If the dataset is balanced, we will jump to the feature selection process.) All the features are not likely equally important for the ML models and sometimes unnecessary features may create complexities in models, resulting in poor performance. Analyzing the characteristics of the dataset, we have selected the ANOVA F-values to extract the important features as described in the Section 3.3. In the next step, we have generated the trained and test sets for model training and evaluation.

### 4.2. Training and Testing Set Generation

We have carefully crafted the training and testing data. From our dataset, we have used 80% data for training and 20% data for testing purposes. During the split of the train and test set, we run a random shuffling and select the test data randomly to reduce the bias of the models. The training dataset has 15 features, with a target feature *Is_Malicious (0→non−malicious and 1→malicious)*. With the training set, we have trained the different classification ML models, including our proposed ensemble model (which will be discussed in the next subsection), and the testing set has been used for evaluating the models’ performance.

### 4.3. Proposed Ensemble Classification Model

We have proposed an ensemble predictive classifier model in combination with five standard classification algorithms. Algorithms are Logistic Regression (LR), Gaussian Naive Bayes (GNB), Support Vector Machine (SVM), K-Nearest Neighbours (KNN), and Decision Tree (DT). A short description of the models is given below.

– **Logistic Regression (LR):** Logistic Regression is a popular machine learning classification model for solving binary classification problems. It finds a relationship between the independent variables and the dependent (target) variable. With the help of the sigmoid function, it represents the probability of the occurrence of an event. In order to calculate the probability of observed data, it optimizes coefficients for independent variables. The impact of each feature is determined by the optimized coefficients of the independent variables [30].
– **Gaussian Naive Bayes (GNB):** Gaussian Naive Bayes is a simple but effective machine learning classification algorithm. It considers that each independent feature is represented with Gaussian distribution for individual classes. The probability of a class is calculated using the Gaussian probability distribution for each feature based on the Naive Bayes theorem. It performs well for datasets where the features are continuous [31].
– **K-Nearest Neighbours (KNN):** KNN is the simplest classifier algorithm but is effective for solving simple problems. It calculates the distance of all the training datapoints from the new datapoint that we want to predict. Finally, it considers the K number of nearest datapoints around the new datapoint and assigns the majority class to the new datapoint [32].
– **Linear Support Vector Machine (SVM):** SVM is a binary classification algorithm. SVM finds the optimal decision boundary (hyperplane) between two different classes. In the training phase, the algorithm tries to maximize the margin between the classes. Thus, it finds the optimal hyperplane. Any new instance is classified based on the decision boundary [33].
– **Decision Tree (DT):** DT is a tree-based predictive model. The data are partitioned recursively according to the most informative features to build the tree. The decision tree consists of nodes, branches, and leaves where nodes represent decision points, branches represent possible choices, and leaves represent outcomes [34].

*We have individually trained all the classifier algorithms and used them to predict the output for each instance of the testing dataset. The predicted results for each testing dataset have been stored in a list and the final predicted output has been chosen based on the maximum number of votes.* Ensemble learning portion of the methodology (Figure 8) illustrates the process. The entire process has been compiled in Algorithm 2. Finally, we have evaluated our proposed model on the testing dataset.
**Algorithm 2** Ensemble Malicious Node Classifier1:**Input**: A balanced dataset D.2:**Output**: Best performing trained predictive model.3:**procedure** Proposed Ensemble Model(Input)4:    Train Logistic Regression (LR), Gaussian Naive Bayes (GNB), Support Vector Machine (SVM), K-Nearest Neighbours (KNN), and Decision Tree (DT) classifiers individually.5:    Predict test data with the five trained models and store the results in a list.6:    Final predicted class will be selected based on maximum voting.7:    Evaluate the performance of the model.8:**end procedure**

### 4.4. Technique for Explainability Analysis

The method by which ML algorithms produce output seems to be similar to that of a black box. We feed data for training and the model trains its parameters according to the dataset and produces output. An explainability analysis of a machine learning model helps to understand how the model produces outputs and which features contribute to those outputs. In the field of cybersecurity, relying solely on a black box prediction model is not sufficient to prevent cyber attacks [35]. Therefore, we conducted a thorough explainability analysis of our model to ensure its effectiveness [36].

There are some methods for explainability analysis and SHAP (SHapley Additive exPlanations) is one of the most effective methods for interpreting ML models [37]. It is a game-theoretic approach for explaining the output of a machine learning model. The explanation can be discussed both for specific output (local explainability) and overall output (global explainability). We have conducted both explainability analyses and included the results in the Section 6.

## 5. Evaluation and Experimental Analysis

Evaluation of a model and comparison with other models are vital steps in research work to validate its acceptability. In this section, we will present our findings scientifically and include an analysis to explain our model.

We extensively evaluated our proposed models from multiple aspects. For evaluation purposes, we didn’t rely only on the accuracy of the models. We also measured the other benchmark metrics like Precision, Recall, and F1 score to confirm that our model worked well for classifying both Malicious and Non-Malicious sensor nodes. To evaluate our model, we considered Logistic Regression (LR), Gaussian Naive Bayes (GNB), Support Vector Machine (SVM), Decision Tree (DT), Random Forest (RF), XGBoost (XGB), Artificial Neural Network (ANN), 1D Convolutional Neural Network (1D CNN), Recurrent Neural Network (RNN), Long Short-Term Memory (LSTM), and our Proposed Ensemble Learning algorithms. As our malicious sensor node dataset was imbalanced and had an impact on the ML model’s output, we balanced the dataset with our proposed data balancing method and experimented with the actual dataset (without data balancing) and standard data balancing techniques.

### 5.1. Evaluation Metrics

A brief description of some of the evaluation metrics terminologies is given below.

**True Positive (TP):** TP is the outcome of the model’s correct prediction of the positive (e.g., malicious) class.
**False Positive (FP):** FP occurs when the model predicts the negative (e.g., non-malicious) class as a positive class.
**True Negative (TN):** TN is the outcome of the model’s correct prediction of the negative class.
**False Negative (FN):** FN occurs when the model predicts the positive class as a negative class.
**Precision:** Precision is the proportion of true positive (TP) prediction over the total number of positive predictions (TP + FP). Precision is important where the output of the positive class is crucial.
  $$\mathrm{Precision}=\frac{\mathrm{TP}}{\mathrm{TP}+\mathrm{FP}}$$
**Recall:** Recall is the proportion of true positive (TP) prediction over the actual positive class (TP + FN). It is crucial for the application where the cost of false positives is very high.
  $$\mathrm{Recall}=\frac{\mathrm{TP}}{\mathrm{TP}+\mathrm{FN}}$$
**F1-Score:** F1-score is the trade-off between precision and recall, and the harmonic mean of precision and recall. It is important for imbalanced datasets because a model trained on an imbalanced dataset might perform well for one class and poorly for another class. In that case, the F1-score considers both precision and recall value for performance evaluation.
  $$F1\;\mathrm{Score}=\frac{2*\mathrm{Precision}*\mathrm{Recall}}{\mathrm{Precision}+\mathrm{Recall}}$$
**Accuracy:** Accuracy is the metric to measure the overall performance of a classification algorithm. It is the ratio of total correction prediction to the total number of testing samples.
  $$\mathrm{Accuracy}=\frac{\mathrm{TP}+\mathrm{TN}}{\mathrm{TP}+\mathrm{TN}+\mathrm{FP}+\mathrm{FN}}$$

For the evaluation of the models, we have used the above metrics and selected the best model based on the performance.

### 5.2. Model Evaluation without Data Balancing

Our dataset consists of 5% malicious instances and 95% non-malicious instances. The results for the malicious sensor node dataset are presented in Table 4, which shows the precision, recall, F1 score, and accuracy of the 12 machine learning models without applying any data balancing techniques. Unfortunately, most of the models did not perform well in terms of precision, recall, and F1 score. While the proposed ensemble model had high accuracy (97.8%), its F1 score and recall value were below the acceptable range. Additionally, the LR, RF, RNN, and LSTM models were underfitted, indicating that the dataset was not good enough to optimize the parameters during the models’ training period.

### 5.3. Model Evaluation with Random under and SMOTE over Sampled Data

In this subsection, we present the precision, recall, F1 score, and accuracy of ML models trained on random undersampled majority class and SMOTE oversampled minority class datasets. Table 5 shows that the ML models on the traditional data balancing technique have increased the overall performance of the ML models significantly. LR and ANN models still exhibit poor performance. However, our proposed model outperforms other models in terms of performance metrics.

### 5.4. Model Evaluation with MSMOTE and ADASYN Data Balancing Technique

MSMOTE and ADASYN stand for Modified Synthetic Minority Over-sampling Technique and Adaptive Synthetic Sampling, respectively. Both of the models are effective for generating balancing data. MSMOTE modifies the way synthetic samples are generated compared to SMOTE [38]. On the other hand, ADASYN provides more attention where the data are sparse to create synthetic data [39]. We have created balanced data with MSMOTE, and ADASYN and trained the ML models with the datasets. Table 6 illustrates the ML models’ results on both balanced datasets. ADASYN performed better than MSMOTE in terms of the result of evaluation metrics and it is better than the previous methods. Our proposed ensemble method, GNB, SVM, and KNN showed the best performance, achieving around 97% accuracy on the balanced dataset with ADASYN.

### 5.5. Model Evaluation with Proposed Data Balancing Technique

We have proposed a data balancing technique by cluster-based undersampling and SMOTE-based oversampling methods, as described in Section 3.4. With the proposed data balancing technique, most of the algorithms’ precision, recall, F1 score, and accuracy are more than 98%. Though most of the models show excellent performance on the balanced dataset, our proposed ensemble classifier model is exceptional, having a recall value of 1 and an accuracy of 99.7%. These results are compiled in Table 7.

### 5.6. ROC Curve and AUC Score Analysis of the Models

**Figure 9:**
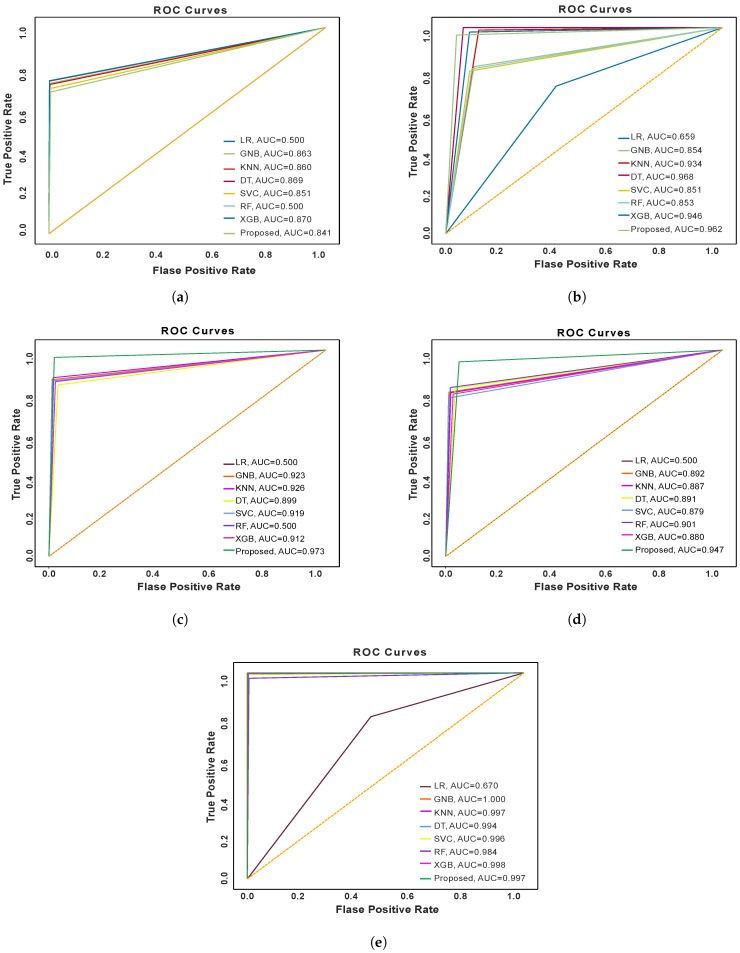
ROC curve and AUC score of the ML models for (**a**) imbalanced dataset, (**b**) balanced dataset with traditional method, (**c**) balanced dataset with ADASYN method, (**d**) balanced dataset with MSMOTE and (**e**) balanced dataset with proposed method.

ROC (Receiver Operating Curve) represents the performance of a classification model at different threshold values in terms of True Positive and False Positive Rate. The AUC score represents the total area under the ROC curve, ranging from [0, 0] to [1, 1] [40]. A higher AUC score indicates a better-performing model. Figure 9 demonstrates the ROC curve analysis of different ML models for three datasets (imbalanced dataset, balanced dataset with traditional techniques, and proposed technique).

Figure 9a shows the result of the different ML models’ ROC curves on the imbalanced dataset. The performance of the Logistic Regression (LR) and Random Forest (RF) models is the poorest, akin to that of a random model, with an AUC score of 0.5. The performance of other models is also unsatisfactory. In Figure 9b, we have represented the ROC curves for ML models trained on the traditional random under-sampling and SMOTE oversampling balanced dataset. The curves demonstrate that the performance has been significantly improved on the balanced dataset. However, Logistic Regression still underperforms on the dataset. The decision tree and our proposed ensemble model show the best performance. Random Forest and Logistic Regression have underperformed on the balanced dataset with ADASYN, but the proposed model outperformed on the dataset, as indicated in Figure 9c. Figure 9d represents the ROC curves for the balanced dataset with the MSMOTE method. Again the Logistic Regression has failed to optimize its parameters and our proposed model performed well. The last Figure 9e illustrates the ROC curves for ML models on the balanced dataset created with our proposed data balancing technique. We have found the highest performance of the ML models on this dataset. The Gaussian Naive Bayes algorithm shows the best performance in terms of AUC, and our proposed model also shows better performance.

### 5.7. Precision vs. Recall Graph Analysis of ML Models

**Figure 10:**
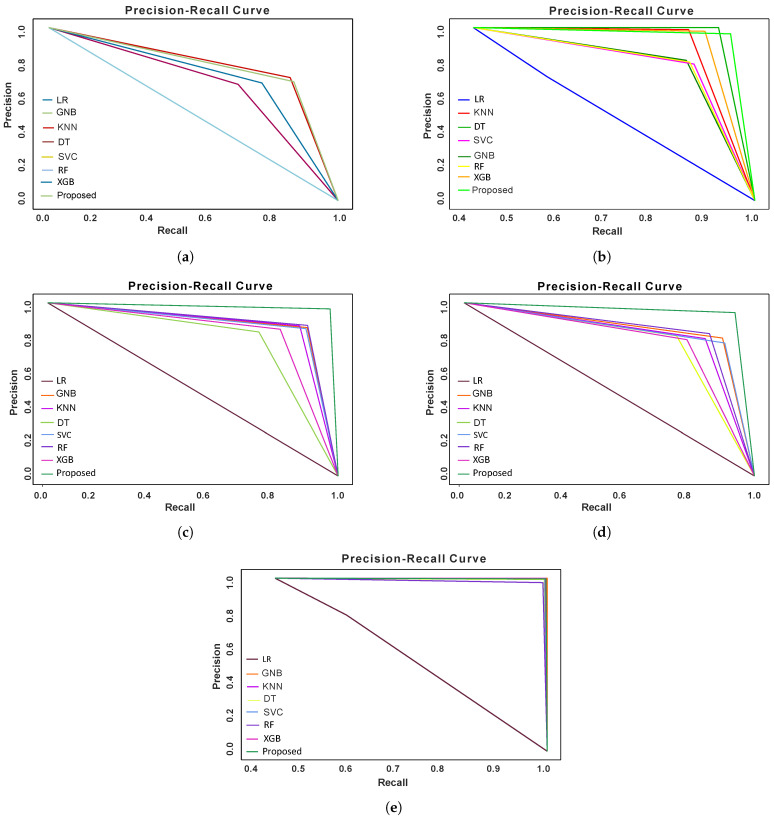
Precision vs. Recall graph of the ML models for (**a**) imbalanced dataset, (**b**) balanced dataset with traditional method, (**c**) balanced dataset with ADASYN, (**d**) balanced dataset with MSMOTE and (**e**) balanced dataset with proposed method.

The Precision vs. Recall graph illustrates how a classification ML model’s precision value changes with the increase of the recall value or vice-versa. A high recall value indicates a model is performing well for positive class (malicious node) identification and it is important for safeguarding cybersecurity. Figure 10 represents the Precision vs. Recall graphs for ML models for three datasets (imbalanced dataset, balanced dataset with traditional techniques, MSMOTE, ADASYN, and proposed technique).

Figure 10a shows the Precision vs. Recall values of the ML models on the imbalanced dataset. The Logistic Regression model’s performance is the worst and other models’ performance for identifying the malicious sensor nodes is also poor. However, KNN and our proposed model’s performance is relatively better than the other models. In Figure 10b, the graph illustrates the Precision vs. Recall graphs for the ML models on the traditional balanced dataset. We see that the Decision Tree model shows the best performance, and the proposed model also performs well. Figure 10c depicts the Precision vs. Recall graph for the ML models trained on the dataset balanced with ADASYN. Our proposed model outperforms the other models and logistics regression is the worst model. We have also generated a Precision vs. Recall graph for the ML models trained on the dataset balanced with the MSMOTE technique represented in Figure 10d. Finally, Figure 10e indicates that all the models show excellent performance except Logistic Regression and in this case, all the models were trained on the dataset created with our proposed data balancing technique.

### 5.8. Overall Comparison

In the previous subsections, we have compared our models in terms of precision, recall, F1 score, accuracy, AUC score, and Precision vs. Recall graphs. We conclude that most of the models show excellent performance on our balanced dataset (with cluster undersampling and SMOTE oversampling), and our proposed ensemble model outperforms most of the ML models. We will show the overall comparison of the models with the F1 score (F1 score is the trade-off between precision and recall) and accuracy on the three datasets.

**Figure 11:**
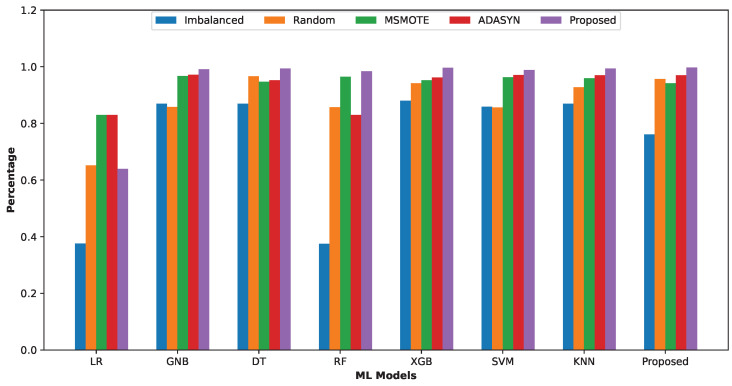
Group (Imbalanced, Traditional Random Balanced, ADASYN Balanced, MSMOTE Balanced and Proposed Balanced Datasets) bar chart based on F1 score for ML models.

Figure 11 exhibits that most of the models’ F1 scores are relatively low for the imbalanced dataset. The F1 score significantly increases when trained with the traditional, ADASYN, and MSMOTE balanced datasets, with the highest F1 score achieved for the balanced dataset created using the proposed method.

**Figure 12:**
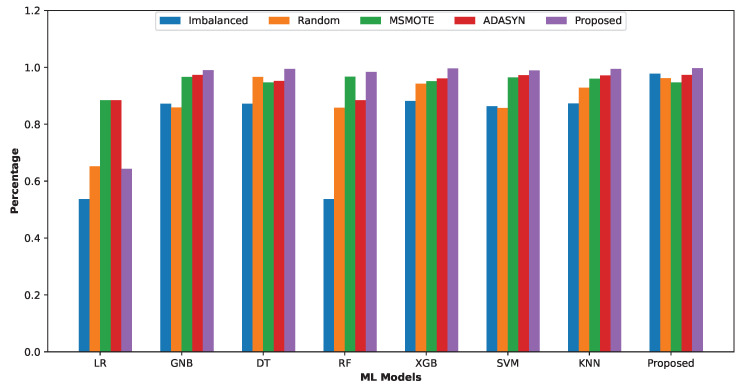
Group (Imbalanced, Traditional Random Balanced, ADASYN Balanced, MSMOTE Balanced and Proposed Balanced Datasets) bar chart based on accuracy for ML models.

Figure 12 illustrates the models’ accuracy on the five types of datasets. From the figure, we observed that all the ML models outperform on the dataset crafted with our proposed data balancing technique. Overall, the balanced dataset improves the ML models’ performance significantly, and our proposed balanced dataset is the best for training models. Additionally, the proposed ensemble classification model shows robustness for all five datasets.

## 6. Explainability Analysis

We have used SHAP to analyze the explainability of our proposed model. SHAP sets a value for each feature in a game-theoretic approach based on its importance for the model’s output value. This section will discuss both local and global explainability.

### 6.1. Local Explainability Analysis

**Figure 13:**
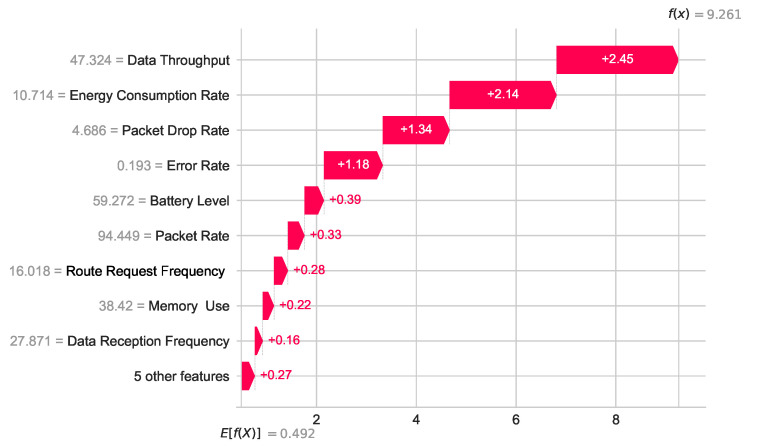
Waterfall plot for the first prediction outcome of the test data.

**Figure 14:**
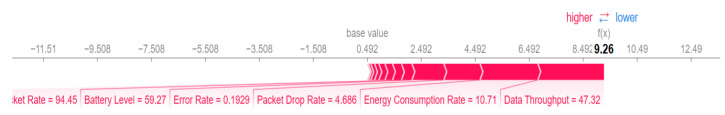
Force plot for the first prediction outcome of the test data.

Local explainability analysis explains the predicted outcome for each test case. Figure 13 illustrates the contribution of each feature to produce the result of the final prediction for the first test dataset, which is a malicious sensor node (class 1). To represent the feature’s importance, SHAP uses log-odd values. E[f(x)]=0.492 is the mean value of the model in the log-odd scale, and f(x)=9.261 is the log-odd value for the first test case. If we add all the feature importance values with the mean value (0.492), we will get the prediction value 9.261. Figure 14 shows how each feature contributes to predicting the output (9.26). For the first test instance, Data Throughput, Energy Consumption Rate, and Packet Drop Rate are the three most important features that lead to the predicted output and other features are also positively contributing to the prediction. Specific feature values have been shown with gray text at the beginning of each feature name.

We can convert the log-odd value to the classification value with the sigmoid function. For demonstration purposes, we consider the log-odd value for the first instance of our test data, which is *f(x)* = 9.261 shown in Figure 13 and Figure 14.

(1) $$\sigma \left(x\right)=\frac{1}{1+e^{-x}}=\frac{1}{1+e^{-9.261}}=1.00$$

Equation (1) explains how the predicted log-odd value can be converted to the classification value. Here, 1 indicates the output is a malicious class.

**Figure 15:**
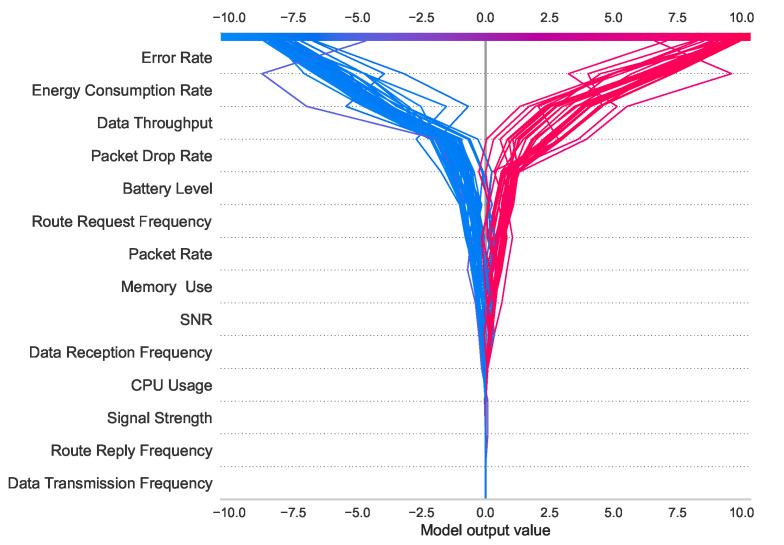
Decision plot of the first 100 test cases of the prediction model.

Figure 15 represents the decision plot for the first hundred test cases. The upper and lower numbers of the *X*-axis [−10 to 10] indicate the log-odd values of the model. The red lines and blue lines symbolize the malicious (class 1) and non-malicious (class 0) classes, respectively. The importance of the features is sorted in descending order from top to bottom.

### 6.2. Global Explanibility Analysis

**Figure 16:**
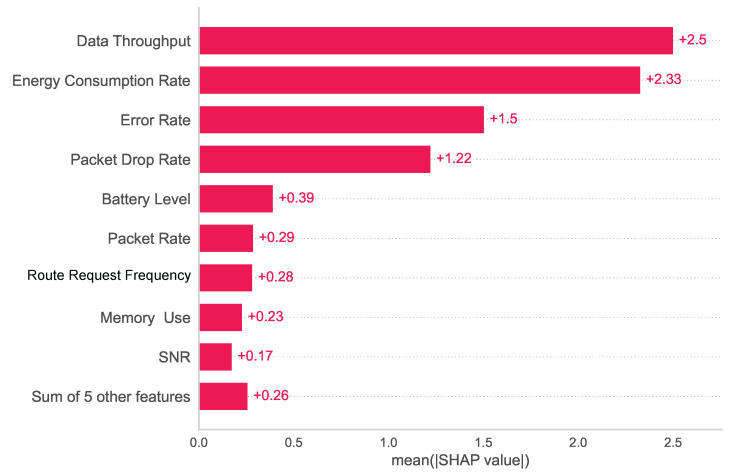
Summary bar plot for the prediction model that represents the overall feature importance of the model.

Figure 16 is generated to show the summary of the global importance (both for malicious and non-malicious classes) of each feature. It considers the absolute mean value for each feature’s importance, the *X*-axis represents the mean SHAP value, and the *Y*-axis represents the feature names in descending order from top to bottom. The last five features have been truncated as these are the least important features.

**Figure 17:**
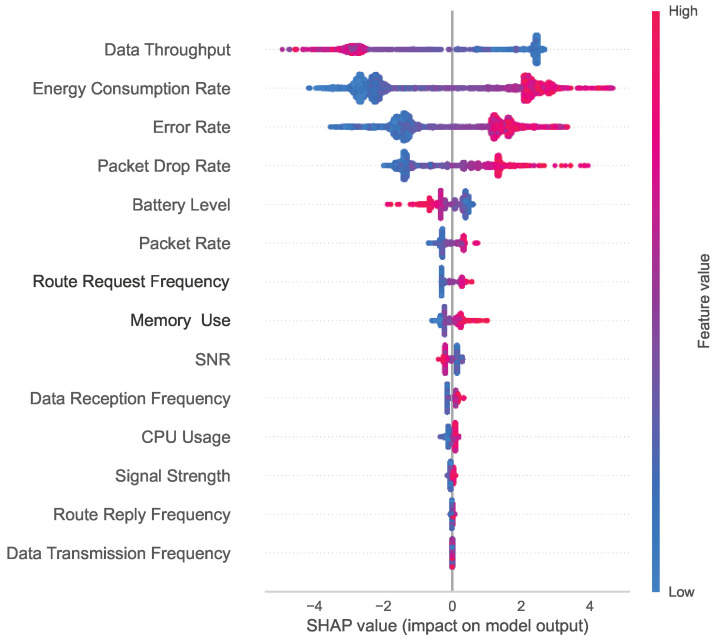
Summary plot for the prediction model showing the importance of each feature along with the feature values.

The summary plot in Figure 17 explicitly shows the importance of each feature with the distribution of the feature values. The vertical line from the 0 value of the *X*-axis is the decision line for the classes where the values on the right side indicate the values of the features for the malicious class and the left side of the values of the features represents the non-malicious class. According to the summary plot, the *Y*-axis represents the feature names and the features are sorted in descending order from top to bottom based on their importance (SHAP values). The color bar at the very right side of the figure represents the color scale for low to high values. Data Throughput is the most important feature of the model. If we closely investigate the feature, we will find a high SHAP value for the negative class with the increase of the feature value, meaning the highest value contributes more to the negative class prediction and vice-versa for the positive class. Additionally, the Data Throughput feature has a high density in the lower value range. All the other features can be described in a similar way.

Overall, the explainability analysis of our proposed model clearly illustrates which features are important for our prediction and how the prediction is being made inside the model. It provides a clear concept of the model’s output.

## 7. Discussion

The aim of this study is to develop an automated system that can detect and prevent malicious sensor nodes in CPS used in various industries. Wireless sensors play a critical role in CPS and IoT technologies. In this research, we have used a dataset derived from IoT components of a CPS. Any attack on the wireless sensors can lead to serious damage to the entire system. Therefore, we have proposed a robust machine learning model that can accurately detect malicious sensor nodes, especially in IoT devices, with an accuracy rate of 99.7%. During our implementation of the model, we faced some challenges because the dataset was highly imbalanced. So, no ML classification model shows desirable performance. We contributed to proposing a data balancing technique and also proposed an ensemble classifier model that outperformed other state-of-the-art ML and Deep Learning (DL) models.

Our proposed classifier achieved 97.8% accuracy on an imbalanced dataset; however, the precision, recall, and F1 scores were below acceptable levels compared to other models. This implies that our model was not effective in detecting malicious sensor nodes because the training dataset lacked positive class data, leading to poor parameter optimization and an unreliable model. So, we balanced the dataset with the traditional method (random undersampling of the majority class and SMOTE oversampling of the minority class). This time the performance of our model was improved significantly by achieving the precision, recall, F1 score, and accuracy of 95.2%, 96.3%, 95.7%, and 96.2%. Though the accuracy decreased by 1.6%, the model’s performance for detecting malicious nodes increased by around 20%. In the case of data balancing techniques with MSMOTE and ADASYN, we found precision, recall, f1-score, and accuracy of 94%, 94.3%, 94.1%, 94.7% and 97.5%, 96.4%, 97%, 97.3%, respectively. When we tested the proposed ensemble model with our balanced dataset created with our proposed data balancing technique, the model showed outstanding precision, recall, F1 score, and accuracy (99.4%, 100%, 99.7%, and 99.7%). After conducting an extensive explainability analysis of our model using SHAP values, we were able to gain a clear understanding of how it produced output at both local and global levels. As a result, the model is now capable of detecting malicious nodes with a high degree of accuracy.

Although the model has demonstrated remarkable performance, the model has been tested with a single dataset to verify the performance of our proposed model. In the future, we will collect identical datasets and test our model’s performance from different dimensions. Furthermore, we intend to develop a model that can detect and correct malicious sensor nodes.

## 8. Conclusions

In this research work, we proposed a robust ensemble learning classifier model that achieved 99.7% accuracy with a high precision and recall value. Our proposed data balancing technique significantly improved the model’s performance by helping to fit the parameters perfectly during the training period. We proposed the data balancing technique with the combination of the cluster undersampling and SMOTE oversampling techniques. Our study concluded with a thorough explainability analysis of our model, providing a comprehensive understanding of its inner workings. This enabled us to identify the root cause of the malicious sensor node with ease, giving us the confidence to take swift and necessary action to protect the system’s security.

## Figures and Tables

**Table 1:** Number of malicious and non-malicious instances of the dataset.

Class Label	Number of Instances ^1^
Non-Malicious	9513
Malicious	487

^1^ The dataset has a total of 10,000 instances.

**Table 2:** Features names along with the category of the features of the dataset.

Category	Features
General Metrics	Node ID
	Timestamp
	IP Address
Network Traffic Metrics	Packet Rate
	Packet Drop Rate
	Packet Duplication Rate
	Data Throughput
Signal Metrics	Signal Strength
	Signal to Noise Ratio
Power Usage Metrics	Battery Level
	Energy Consumption Rate
Routing Metrics	Number of Neighbors
	Routing Request Frequency
	Routing Reply Frequency
Behavioral Metrics	Data Transmission Frequency
	Data Reception Frequency
	Error Rate
Miscellaneous Metrics	CPU Usage
	Memory Usage
	Bandwidth
Metrics Specific to Attacks	Is Malicious

**Table 3:** Some sample instances of the dataset with various attributes.

Node ID	Timestamp	IP Address	Packet Rate	Packet Drop Rate	……	Bandwidth	Is Malicious
1	01-02-23 0:00	192.168.119.138	52.018229	2.727317	……	76.811986	0
2	01-02-23 0:01	192.168.225.56	59.504648	1.435058	……	112.495912	0
15	01-02-23 0:14	192.168.133.9	72.790914	3.803897	……	102.082282	1
78	01-02-23 1:17	192.168.148.225	85.585024	4.038405	……	105.623986	1

**Table 4:** Precision, Recall, F1 score and Accuracy of the ML models trained on the original dataset (without data balancing).

Category	Algorithm	Precision	Recall	F1 Score	Accuracy
ML Models	LR	0.751	0.537	0.376	0.537
	GNB	0.894	0.872	0.869	0.872
	DT	0.894	0.872	0.869	0.872
	RF	0.751	0.537	0.375	0.537
	XGB	0.901	0.882	0.880	0.882
	SVM	0.888	0.863	0.859	0.863
	KNN	0.893	0.871	0.869	0.873
DL Models	ANN	0.872	0.834	0.826	0.834
	1D CNN	0.820	0.841	0.831	0.840
	RNN	0.762	0.641	0.696	0.782
	LSTM	0.752	0.541	0.380	0.541
**Proposed**	**Proposed**	**0.854**	**0.687**	**0.761**	**0.978**

**Table 5:** Precision, Recall, F1 score, and Accuracy of the ML models trained on random undersampled and SMOTE oversampled balanced data.

Category	Algorithm	Precision	Recall	F1 Score	Accuracy
ML Models	LR	0.664	0.652	0.652	0.652
	GNB	0.859	0.859	0.858	0.859
	DT	0.966	0.968	0.966	0.966
	RF	0.859	0.858	0.857	0.858
	XGB	0.942	0.945	0.942	0.942
	SVM	0.859	0.857	0.856	0.857
	KNN	0.928	0.935	0.928	0.928
DL Models	ANN	0.698	0.673	0.671	0.673
	1D CNN	0.862	0.891	0.876	0.901
	RNN	0.810	0.831	0.820	0.862
	LSTM	0.824	0.825	0.824	0.825
**Proposed**	**Ensemble**	**0.952**	**0.963**	**0.957**	**0.962**

**Table 6:** Precision, Recall, F1 score, and Accuracy of the ML models trained on MSMOTE and ADASYN balanced datasets.

Category	Algorithm	MSMOTE				ADASYN			
		Pre.	Rec.	F1	Acc.	Pre.	Rec.	F1	Acc.
ML Models	LR	0.898	0.884	0.830	0.884	0.898	0.884	0.830	0.884
	GNB	0.965	0.966	0.965	0.966	0.972	0.973	0.972	0.973
	DT	0.948	0.946	0.947	0.946	0.953	0.951	0.952	0.951
	RF	0.965	0.966	0.965	0.966	0.898	0.884	0.830	0.884
	XGB	0.951	0.951	0.951	0.951	0.961	0.961	0.961	0.961
	SVM	0.963	0.964	0.963	0.964	0.971	0.972	0.971	0.972
	KNN	0.959	0.960	0.959	0.960	0.971	0.971	0.971	0.971
DL Models	ANN	0.898	0.850	0.873	0.883	0.888	0.923	0.902	0.912
	1D CNN	0.931	0.943	0.937	0.949	0.960	0.931	0.945	0.955
	RNN	0.902	0.898	0.900	0.910	0.919	0.901	0.910	0.922
	LSTM	0.930	0.945	0.937	0.969	0.931	0.922	0.926	0.938
**Proposed**	**Ensemble**	**0.940**	**0.943**	**0.941**	**0.947**	**0.975**	**0.964**	**0.970**	**0.973**

**Table 7:** Precision, Recall, F1 score, and Accuracy of the ML models trained on proposed cluster undersampled and SMOTE oversampled balanced dataset.

Category	Algorithm	Precision	Recall	F1 Score	Accuracy
ML Models	LR	0.666	0.643	0.639	0.643
	GNB	0.992	0.992	0.991	0.990
	DT	0.995	0.994	0.994	0.994
	RF	0.985	0.984	0.984	0.984
	XGB	0.997	0.996	0.996	0.996
	SVM	0.984	0.993	0.988	0.989
	KNN	0.995	0.994	0.994	0.994
DL Models	ANN	0.852	0.846	0.845	0.846
	1D CNN	0.952	0.895	0.923	0.961
	RNN	0.962	0.948	0.955	0.959
	LSTM	0.973	0.945	0.959	0.968
**Proposed**	**Ensemble**	**0.994**	**1.0**	**0.997**	**0.997**

## Data Availability

Data is publicly available at IEEE DataPort.

## References

[1] Colombo A.W., Karnouskos S., Kaynak O., Shi Y., Yin S. (2017). Industrial cyberphysical systems: A backbone of the fourth industrial revolution. IEEE Ind. Electron. Mag..

[2] Kayan H., Nunes M., Rana O., Burnap P., Perera C. (2022). Cybersecurity of industrial cyber-physical systems: A review. ACM Comput. Surv..

[3] Javaid M., Haleem A., Rab S., Singh R.P., Suman R. (2021). Sensors for daily life: A review. Sens. Int..

[4] Boubiche D.E., Athmani S., Boubiche S., Toral-Cruz H. (2021). Cybersecurity issues in wireless sensor networks: Current challenges and solutions. Wirel. Pers. Commun..

[5] Duobiene S., Ratautas K., Trusovas R., Ragulis P., Šlekas G., Simniškis R., Račiukaitis G. (2022). Development of wireless sensor network for environment monitoring and its implementation using SSAIL technology. Sensors.

[6] Apruzzese G., Laskov P., Montes de Oca E., Mallouli W., Brdalo Rapa L., Grammatopoulos A.V., Di Franco F. (2023). The role of machine learning in cybersecurity. Digit. Threat. Res. Pract..

[7] Raghunath K.M.K., Arvind K.S. (2023). SensorNetGuard: A Dataset for Identifying Malicious Sensor Nodes. IEEEDataPort.

[8] Sarker I.H. (2024). AI-Driven Cybersecurity and Threat Intelligence: Cyber Automation, Intelligent Decision-Making and Explainability.

[9] Mokhtar R., Rohaizat A. (2024). Cybercrimes and cyber security trends in the new normal. The New Normal and Its Impact on Society: Perspectives from ASEAN and the European Union.

[10] Sarker I.H. (2023). Multi-aspects AI-based modeling and adversarial learning for cybersecurity intelligence and robustness: A comprehensive overview. Secur. Priv..

[11] Makanju A., LaRoche P., Zincir-Heywood A.N. (2024). A Comparison between Signature and Machine Learning Based Detectors.

[12] Tan X., Su S., Huang Z., Guo X., Zuo Z., Sun X., Li L. (2019). Wireless sensor networks intrusion detection based on SMOTE and the Random Forest algorithm. Sensors.

[13] Wang W., Huang H., Li Q., He F., Sha C. (2020). Generalized intrusion detection mechanism for empowered intruders in wireless sensor networks. IEEE Access.

[14] Whelan J., Sangarapillai T., Minawi O., Almehmadi A., El-Khatib K. Novelty-based intrusion detection of sensor attacks on unmanned aerial vehicles. Proceedings of the 16th ACM Symposium on QoS and Security for Wireless and Mobile Networks.

[15] Ding H., Chen L., Dong L., Fu Z., Cui X. (2022). Imbalanced data classification: A KNN and generative adversarial networks-based hybrid approach for intrusion detection. Future Gener. Comput. Syst..

[16] Fu Y., Du Y., Cao Z., Li Q., Xiang W. (2022). A deep learning model for network intrusion detection with imbalanced data. Electronics.

[17] Moundounga A.R.A., Satori H., Boutazart Y., Abderrahim E. (2023). Malicious attack detection based on continuous Hidden Markov Models in Wireless sensor networks. Microprocess. Microsyst..

[18] Saleh H.M., Marouane H., Fakhfakh A. (2024). Stochastic Gradient Descent Intrusions Detection for Wireless Sensor Network Attack Detection System Using Machine Learning. IEEE Access.

[19] Salmi S., Oughdir L. (2023). Performance evaluation of deep learning techniques for DoS attacks detection in wireless sensor network. J. Big Data.

[20] Almomani I., Al-Kasasbeh B., Al-Akhras M. (2016). WSN-DS: A dataset for intrusion detection systems in wireless sensor networks. J. Sens..

[21] Taher M.A., Iqbal H., Tariq M., Sarwat A.I. Recurrent neural network—Based sensor data attacks identification in distributed renewable energy—Based DC microgrid. Proceedings of the 2024 IEEE Texas Power and Energy Conference (TPEC).

[22] Nouman M., Qasim U., Nasir H., Almasoud A., Imran M., Javaid N. (2023). Malicious node detection using machine learning and distributed data storage using blockchain in WSNs. IEEE Access.

[23] Hasan M., Rahman M.S., Janicke H., Sarker I.H. (2024). Detecting Anomalies in Blockchain Transactions using Machine Learning Classifiers and Explainability Analysis. arXiv.

[24] Kilkenny M.F., Robinson K.M. (2018). Data quality: Garbage in–garbage out. Health Inf. Manag. J. Health Inf. Manag. Assoc. Aust..

[25] Van der Maaten L., Hinton G. (2008). Visualizing data using t-SNE. J. Mach. Learn. Res..

[26] Elssied N.O.F., Ibrahim O., Osman A.H. (2014). A novel feature selection based on one-way anova f-test for e-mail spam classification. Res. J. Appl. Sci. Eng. Technol..

[27] Humaira H., Rasyidah R. Determining the appropiate cluster number using elbow method for k-means algorithm. Proceedings of the 2nd Workshop on Multidisciplinary and Applications (WMA).

[28] Zubair M., Iqbal M.A., Shil A., Chowdhury M., Moni M.A., Sarker I.H. (2022). An improved K-means clustering algorithm towards an efficient data-driven modeling. Ann. Data Sci..

[29] Chawla N.V., Bowyer K.W., Hall L.O., Kegelmeyer W.P. (2002). SMOTE: Synthetic minority over-sampling technique. J. Artif. Intell. Res..

[30] Hosmer D.W., Lemeshow S., Sturdivant R.X. (2013). Applied Logistic Regression.

[31] Reddy E.M.K., Gurrala A., Hasitha V.B., Kumar K.V.R. (2022). Introduction to Naive Bayes and a review on its subtypes with applications. Bayesian Reasoning and Gaussian Processes for Machine Learning Applications.

[32] Géron A. (2022). Hands-on Machine Learning with Scikit-Learn, Keras, and TensorFlow.

[33] Hearst M.A., Dumais S.T., Osuna E., Platt J., Scholkopf B. (1998). Support vector machines. IEEE Intell. Syst. Their Appl..

[34] Song Y.Y., Ying L. (2015). Decision tree methods: Applications for classification and prediction. Shanghai Arch. Psychiatry.

[35] Sarker I.H., Janicke H., Mohsin A., Gill A., Maglaras L. (2024). Explainable AI for cybersecurity automation, intelligence and trustworthiness in digital twin: Methods, taxonomy, challenges and prospects. ICT Express.

[36] Linardatos P., Papastefanopoulos V., Kotsiantis S. (2020). Explainable ai: A review of machine learning interpretability methods. Entropy.

[37] Lundberg S.M., Lee S.I. A unified approach to interpreting model predictions. Proceedings of the Advances in Neural Information Processing Systems 30 (NIPS 2017): 31st Annual Conference on Neural Information Processing Systems.

[38] Hu S., Liang Y., Ma L., He Y. MSMOTE: Improving classification performance when training data is imbalanced. Proceedings of the IEEE 2009 s International Workshop on Computer Science and Engineering.

[39] He H., Bai Y., Garcia E.A., Li S. ADASYN: Adaptive synthetic sampling approach for imbalanced learning. Proceedings of the 2008 IEEE International Joint Conference on Neural Networks (IEEE World Congress on Computational Intelligence).

[40] Bradley A.P. (1997). The use of the area under the ROC curve in the evaluation of machine learning algorithms. Pattern Recognit..

